# SOAT1 regulates cholesterol metabolism to induce EMT in hepatocellular carcinoma

**DOI:** 10.1038/s41419-024-06711-9

**Published:** 2024-05-09

**Authors:** Rongrong Fu, Wenqing Xue, Jingjie Liang, Xinran Li, Juan Zheng, Lechen Wang, Min Zhang, Jing Meng

**Affiliations:** 1 State Key Laboratory of Food Nutrition and Safety, College of Food Science and Engineering, Tianjin University of Science and Technology, Tianjin, China; 2 Joint Laboratory for Translational Medicine Research, Liaocheng People’s Hospital, Liaocheng, China; 3 China-Russia Agricultural Products Processing Joint Laboratory, Tianjin Agricultural University, Tianjin, China; 4 Tianjin International Joint Academy of Biomedicine, Tianjin, China

**Keywords:** Cancer metabolism, Cell signalling

## Abstract

Cholesterol metabolism reprogramming is one of the significant characteristics of hepatocellular carcinoma (HCC). Cholesterol increases the risk of epithelial–mesenchymal transition (EMT) in cancer. Sterol *O*-acyltransferases 1 (SOAT1) maintains the cholesterol homeostasis. However, the exact mechanistic contribution of SOAT1 to EMT in HCC remains unclear. Here we demonstrated that SOAT1 positively related to poor prognosis of HCC, EMT markers and promoted cell migration and invasion in vitro, which was mediated by the increased cholesterol in plasmalemma and cholesterol esters accumulation. Furthermore, we reported that SOAT1 disrupted cholesterol metabolism homeostasis to accelerate tumorigenesis and development in HCC xenograft and NAFLD-HCC. Also, we detected that nootkatone, a sesquiterpene ketone, inhibited EMT by targeting SOAT1 in vitro and in vivo. Collectively, our finding indicated that SOAT1 promotes EMT and contributes to hepatocarcinogenesis by increasing cholesterol esterification, which is suppressed efficiently by nootkatone. This study demonstrated that SOAT1 is a potential biomarker and therapeutic target in NAFLD-HCC and SOAT1-targeting inhibitors are expected to be the potential new therapeutic treatment for HCC.

## Introduction

Hepatocellular carcinoma (HCC) is one of the most leading causes of cancer in the worldwide with high mortality rate [1]. Following with the raised incidence of non-alcoholic fatty liver disease (NAFLD) worldwide, NAFLD-associated HCC (NAFLD-HCC) was increasingly recognized as the leading cause of HCC [2]. Increasing evidence demonstrates that abnormal lipid metabolism is a vital reason for cancer occurrence [3]. Epithelial–mesenchymal transition (EMT) plays an important role in tumor metastasis, in which epithelial cells lose epithelial characteristics and acquire mesenchymal phenotype [4]. EMT can coordinate kinds of complementary cancer characteristics, including tumor cell stemness, tumorigenicity, resistance to therapy, and adaptation to variable microenvironment [5]. Though there are novel chemotherapeutic interventions and target therapy, the overall prognosis of patients with HCC is still poor owe to the high rates of intrahepatic and distal metastasis [6, 7]. Previous studies have reported that EMT can be regulated to promote metastasis in HCC. Cancer stem cells own infinite differentiation potential and self-renewal properties, which lead to cell growth, distant and metastasis in HCC [8, 9]. Therefore, exploring the molecular mechanism of regulating the EMT in HCC cells has great significance for inhibiting the HCC metastasis and improving the poor prognosis of patients.

Cholesterol is an essential lipid of plasma membrane and supports the requirements of cell proliferation growth and structure [10]. Reprogramming cholesterol metabolism such as accelerated synthesis and abnormal uptake are closely related to the development of HCC [11, 12]. In the tumor microenvironment, cholesterol metabolism is usually enhanced to support cancer progression. Squalene epoxidase (SQLE) induced the development of NAFLD-HCC via promoting the biosynthesis of cholesterol esters [13]. Cholesterol uptake through the low-density lipoprotein receptor (LDLR) is vital for the growth of ALK+ anaplastic large cell lymphoma cells and patient-derived xenografts [12]. In addition, dietary cholesterol has been proved to promote NAFLD-HCC [14]. 3-Hydroxy-3-methylglutaryl-CoA reductase (HMGCR) expression was up-regulated in human HCC along with enhanced mitochondrial cholesterol content [15]. 3-hydroxy-3-methylglutaryl CoA synthase (HMGCS) can convert acetyl coenzyme A into HMG-CoA, which plays an important role in the process of cholesterol synthesis [16]. CSN6 stabilizes HMGCS1 protein by preventing SPOP-mediated HMGCS1 ubiquitination and degradation [17]. Cholesterol drives the development of HCC by regulating the distribution of lncRNA SNHG6 between organelles [18]. Cholesterol and its metabolites also effect immune microenvironment of tumor. Oxysterols, a cholesterol oxidation product, regulates transcription factors (SREBP2 and LXR) related to cholesterol metabolism, which in turn leads to T cell dysfunction [19]. Therefore, targeting cholesterol metabolism reprogramming could regulate the genesis and metastasis in HCC.

Sterol *O*-acyltransferases (SOAT), also known as ACAT, has two isoforms in mammals, SOAT1 and SOAT2. SOAT1, localized on the endoplasmic reticulum, performs catalytic functions as dimers or tetramers and could esterify cholesterol to cholesterol ester [20]. Increasing evidence indicates that the suppression of SOAT1 blocked tumor growth, such as glioblastoma [21], pancreatic cancer [22], colon cancer [23], and gastric cancer [24]. However, the underlying mechanism whether SOAT1 promotes EMT in HCC remains to be explored.

Terpenoids have been widely studied in vitro and in vivo due to their unique anti-tumor biological characteristics [25]. Nootkatone, a natural plant sesquiterpene ketone, exhibits pharmacological activity on regulating inflammation, apoptosis, and autophagy [26, 27]. Besides, nootkatone exerts anticancer effects on colorectal cancer [27] and non-small-cell-lung cancer [28]. And, it has been reported that nootkatone has effect on hepatic fibrosis [29]. However, the inhibitive effect of nootkatone on HCC has not been reported.

Recently, it has proved that SOAT1 promoted proliferation and is associated with early-stage HCC [30]. However, whether SOAT1 promotes EMT in HCC has not been known yet. Here, we report a direct correlation of increased expression of SOAT1 with EMT in HCC. Our results indicate that SOAT1 promotes EMT in HCC through maintaining cholesterol homeostasis and therapeutic efficacy of nootkatone on NAFLD-HCC, which supports the critical role of the metabolic microenvironment in HCC metastasis, providing a new insight for the precision therapy on HCC.

## Materials and methods

### Bioinformatic analysis

Five gene expression profiles GSE99807 [31], GSE164760 [32], GSE50579 [33], GSE14520-GPL3921 [34], and GSE33006 [35] were obtained from Gene Expression Omnibus (GEO) (https://www.ncbi.nlm.nih.gov/geo/). Differentially expressed genes were screened through GEO2R. Then the up-regulated and down-regulated DEGs were, respectively, intersected via Venn analysis (https://bioinformatics.psb.ugent.be/webtools/Venn/) to obtain mutual DEGs. The Gene Ontology (GO) annotations and the Kyoto Encyclopedia of Genes and Genomes (KEGG) pathway enrichment analysis of DEGs were performed using DAVID 2021 software (https://david.ncifcrf.gov/). The interaction between DEGs were confirmed by STRING database (https://cn.string-db.org/). Representative IHC pictures of SOAT1 were obtained from The Human Protein Atlas (THPA) (https://www.proteinatlas.org/). SOAT1 expression data and clinical data were extracted from The Cancer Genome Atlas (TCGA) database (https://portal.gdc.cancer.gov/). SOAT1 expression in HCC cells was obtained from Cancer Cell Line Encyclopedia (CCLE) (https://sites.broadinstitute.org/ccle/datasets).

### HCC sample analysis

Twenty-two cases of HCC samples were collected from Liaocheng People’s Hospital, which was individually examined by certified pathologists for Edmondson–Steiner (ES) grade and microvascular invasion (MVI) grade according to the WHO published standardizations. This study has been approved by the Ethics Committee of Liaocheng People’s Hospital and Tianjin University of Science and Technology. The sample collection has obtained informed consent from all participants. Immunohistochemistry (IHC) analyzed the expression level of SOAT1 and its correlation with ES and MVI grade.

### Cell culture

HepG2 and PLC/PRF/5 cells were purchased from Cell Bank of Shanghai Institutes for Biological Sciences (Shanghai, China). Cells were cultured in DMEM medium (Gibco) with 10% FBS (YEASEN) and 1% antibiotics (100 U/mL penicillin and 10 μg/mL streptomycin, YEASEN) at 37 °C in an atmosphere of 5% CO_2_.

### Plasmid construction and transfection

pcDNA3.1-3×Flag-SOAT1 and pRNAT-U6.1/Neo was purchased from YouBio. Small hairpin RNA (shRNA) targeting human SOAT1 was constructed into pRNAT-U6.1/Neo. The sequence of shSOAT1 was as follows: (5’-TGGTCCATGACTGGCTATATTCTCGAGAATATAGCCAGTCATGGACCATTTTTT-3’).

Cells were transfected with plasmids DNA at 70–95% confluence using Hieff Trans TM Liposomal transfection Reagent (YEASEN) in accordance with the manufacturer’s instructions.

### Western blot analysis

Cells or tissues were washed by precooling PBS and lysed with cold RIPA buffer (Beyotime) containing protease inhibitor cocktail (MCE) in ice for 30 min, followed by centrifugation at 12,000 rpm for 10 min. The supernatant was collected, and protein concentrations were determined using the BCA Protein Assay Kit (Beyotime). Total protein was separated by SDS-PAGE and transferred onto PVDF membrane (Millipore) and the membranes were blocked with 5% non-fat milk for 1 h. Then, the membrane was incubated over night at 4°C with the following primary antibodies: rabbit anti-SOAT1 (ABclonal, 1:1000), rabbit anti-E-cadherin (Bioss, 1:1000), rabbit anti-Occludin (Proteintech, 1:10,000), rabbit anti-Vimentin (Bioss, 1:1000), rabbit anti-Twist1 (Proteintech, 1:2000), rabbit anti-N-cadherin (Proteintech, 1:5000), rabbit anti-Slug (Proteintech, 1:10,000), rabbit anti-Snail1 (Proteintech, 1:800), rabbit anti-Fibronectin (Proteintech, 1:10,000), rabbit anti-SREBP2 (ABclonal, 1:1000), rabbit anti-LDLR (Proteintech, 1:2000), rabbit anti-ITGB4 (Proteintech, 1:600), rabbit anti-ITGAV (Abbkine, 1:1000), rabbit anti-AFP (Proteintech, 1:1500), mouse anti-GAPDH (Proteintech, 1:25,000). Then, the membranes were washed three times with TBST for 10 min at room temperature and incubated with secondary antibody (YEASEN, 1:5000) at room temperature for 1 h. The bands were visualized using SuperKine™ ECL (Abbkine) with Chemiluminescent Imaging System (Image Quant LAS 4000).

### Immunofluorescence

HepG2 and PLC/PRF/5 cells with different treatments were fixed with 4% paraformaldehyde for 20 min at room temperature and blocked with 5% BSA for 30 min. Cells were incubated at temperature with E-cadherin (Proteintech, 1:200) and Vimentin antibody (Bioss, 1:100) at room temperature for 1 h. Then the cells were treated with secondary antibodies (YEASEN, 1:100) at room temperature for 1 h. The cells were washed twice with PBS and every time for 5 min in each step. Cell slides were sealed with anti-fluorescence quenching sealer with DAPI (YEASEN) and the fluorescence was detected with Laser scanning confocal microscope (ZEISS).

### Scanning electron microscope (SEM)

HepG2 and PLC/PRF/5 cells with different treatments were fixed, dehydrated in gradient concentration of ethanol and dried with the gradient concentration of acetonitrile. Then cells were dried again by vacuum freeze dryer. Gold-plated cells were photographed through scanning electron microscopy (JEOL).

### Wound healing assay

HepG2 or PLC/PRF/5 with different treatments were inoculated in wells for 12 h at 37 °C. The pipettor tips were used to remove part cells in each well. The floating cells were washed off with PBS and then serum-free medium was added. Immediately after, the wound was photographed, which was regarded as 0 h wound distance. After 24 and 48 h of cell culture, the wound was recorded. The migration of cells was analyzed by comparing the wound distance ratio at 0 h.

### Invasion assay

Invasion assays were performed using transwell plates (8μm pore filter, Corning). HepG2 or PLC/PRF/5 cells with different treatments were seeded in top chamber inserts added with Matrigel (BD Biosciences). The upper chamber was added with 200 μL of the cell suspension (2 × 10^5^ cells/mL) in serum-free medium. The bottom chamber was filled with 700 μL of medium containing 20% FBS. After 24 h of incubation, cells located on the lower surface of the chamber were fixed for 10 min with cool methanol. Then cells were stained with crystal violet for 10 min. After being cleaned with PBS, the cells were photographed under the microscope and counted using ImageJ software.

### Cell counting kit-8 (CCK-8) assay

After transfection for 24 h, HepG2 or PLC/PRF/5 cells were seeded in a 96-well plate with a density of 2 × 10^3^/mL in each well. Then cell medium (each well 100 μL) and CCK-8 reagent (Beyotime) were added to detect cell proliferation at 24, 48, and 72 h. The absorbance (*A*) was measured at 450 nm. Five parallel wells were set in each group, and the mean value was obtained. Cell survival rate was calculated using the following formula: cell survival rate (%) = (*A*_experimental group_/*A*_control group_) × 100%.

### Oil red staining

Cells with different treatment were fixed with 4% paraformaldehyde for 10 min. Fresh-frozen tissue sections (8μm) were performed. All staining procedures were carried out according to Modified Oil Red O Staining Kit (Beyotime). Nucleus were stained with hematoxylin (Proteintech) 5 s. Samples were rinsed three times with PBS and photographed by Microscope (Olympus). Quantitative analysis was performed by ImageJ software.

### BODIPY 493/503 staining

Cells with different treatment were fixed with 4% paraformaldehyde for 20 min. After being cleaned with PBS, lipid droplets were stained by 5 μM BODIPY 493/503 (GLPBIO) for 30 min and visualized by Laser scanning confocal microscope (ZEISS). Quantitative analysis was performed by ImageJ Pro software.

### Free cholesterol/cholesterol ester concentrations

Cells (5 × 10^6^) or tissues (50 mg) were harvested and treated according to kit instructions. Free cholesterol concentrations were detected by Free Cholesterol (FC) Quantification Kit (Abbkine). Total cholesterol (TC) concentrations were detected by Total Cholesterol Quantification Kit (Abbkine). Cholesterol esters concentration was the difference between TC concentration and FC concentration.

### Filipin III staining

Cells were fixed with 4% paraformaldehyde for 15 min and incubated with 1.5 mg/mL Glycine for 30 min. Wash cells three times with PBS. Cells were incubated with 12.7 μM Filipin III (GLPBIO) for 2 h. The fluorescence was detected with Laser scanning confocal microscope (ZEISS).

### Molecular docking

The 3D structure of SOAT1 was downloaded from the PDB database (PDB code 6VUM). The 3D structure of nootkatone (NK) was drawn by ChemDraw 2D 20.0 and transformed by Chem 3D. Molecular docking of nootkatone and SOAT1 protein was completed by AutoDockTools [36, 37]. The docking result was beautified through PyMOL.

### Xenograft tumor model

Male BALB/c nude mice (5–6 weeks old) purchased from SPF (Beijing) Biotechnology Co., Ltd were randomly divided into Control, SOAT1, shSOAT1, NK, and SOAT1 + NK groups (*n* = 6 per group). Mice were subcutaneously injected with PLC, PLC-SOAT1, PLC-shSOAT1 cells (1 × 10^7^). When the tumor size was up to 0.2 cm [3], the mice in NK and SOAT1 + NK group were given nootkatone (dissolved in 0.5% CMC-Na) by gavage at 200 mg/kg/day for 18 days. Other groups were given the same volume of 0.5% CMC-Na gastric irrigation. Tumor size was tested and calculated every 3 days according to the standard formula. All mice were sacrificed at the 28th day post inoculation, and the tumor tissues were collected, photographed, and weighted.

### Pulmonary metastasis model

Male BALB/c nude mice (5–6 weeks old) were randomly divided into Control, SOAT1, shSOAT1, NK, and SOAT1 + NK groups (*n* = 6 per group). Mice were injected with PLC, PLC-SOAT1, PLC-shSOAT1 cells (2 × 10^6^) via tail vein. The mice in NK and SOAT1 + NK groups were given nootkatone (dissolved in 0.5% CMC-Na) by gavage at 200 mg/kg for total 4 weeks. Other groups were given the same volume of 0.5% CMC-Na gastric irrigation. After eight weeks, lungs were collected, photographed and metastatic nodules were counted.

### NAFLD-HCC model

Male C57BL/6J (2-week-old) mice were purchased from SPF (Beijing) Biotechnology Co., Ltd. Husbandry was conducted following standard guidelines. After the accommodation, mice were divided randomly into four groups (*n* = 6 per group): control group that fed with normal chow; model group that injected once a week with 25 mg/kg Diethylnitrosamine (DEN, N0258, Sigma) for 2 weeks and then fed with high-fat/high-cholesterol diet (HFHC, TP28522, containing 40% fat and 0.5% cholesterol), lasting 20 weeks. At 19 weeks of age, nootkatone (dissolved in 0.5% CMC-Na) was given by gavage at 100 mg/kg/day (NK-L group) and 200 mg/kg/day (NK-H group). At the same time, model group was given an equivalent volume of 0.5% CMC-Na. At 24 weeks of age, serum and liver tissue were obtained for further analysis. Moreover, part of liver was fixed in 10% neutral formalin for histological analysis. Animal studies were carried out according to the Guidelines of Animal Experimentation Ethics Committee, Tianjin University of Science and Technology.

### Biochemical analysis

Contents of total cholesterol (TC) was quantified by commercial kits (Jiancheng Biotech). Serum AFP was detected with mouse-AFP ELISA kits (mlbio). The levels of hepatic free cholesterol (FC) and cholesterol ester (CE) were determined using FC Quantification Kit (Abbkine) and TC Quantification Kit (Abbkine).

Serum alanine aminotransferase (ALT) and aspartate aminotransferase (AST) in mice were detected by biochemical analyzer (Hitachi 7020).

### Histological analysis

Frozen sections were stained with Oil red O to visualize lipid droplets. The fixed liver tissue embedded in paraffin were sliced (3μm). After dewaxing, tissue samples were stained with hematoxylin–eosin (H&E). Fibrosis was assessed by Sirius Red staining (G-CLONE). All slices were observed using a microscope (Olympus).

### Immunohistochemistry (IHC) staining

Paraffin sections of liver tissue (3 μm) were dewaxed and rehydrated. After being blocked with 3% H_2_O_2_ for 10 min, sections were sealed with goat serum (Proteintech) for 20 min. Then sections were incubated with the following primary antibodies overnight at 4 °C: SOAT1 (BOSTER, 1:200), mouse anti-E-cadherin (Proteintech, 1:200), rabbit anti-Vimentin (Bioss, 1:400). The primary antibody in negative group was replaced by PBS. After washing, horseradish peroxidase-polymer anti-mouse/rabbit (Maixin Biotech) was added in all sections for 1 h at room temperature. Lastly, tissues were stained with 3,3’-diamino-benzidine-tetrahydrochloride and counterstained with hematoxylin (Maixin Biotech). All sections were observed with microscope (Olympus). The IHC score was calculated by multiplying the intensity (negative = 0, canary yellow = 1, claybank = 2, brown = 3) and the positive cell percentage scores (<25% = 1, 25–50% = 2, 51–75% = 3, >75% = 4).

### Real-time quantitative reverse transcription PCR

Total RNA in liver tissue was extracted by Trizol Reagent (TIANGEN Biotech) and converted into cDNA with Reverse Transcription Kit (TIANGEN Biotech). The method of RT-PCR was according to the manufacturer’s instructions and previously described protocol [38]. GAPDH expression was regarded as standard.

### mRNA sequencing and bioinformation analysis

Total RNA was extracted, purified, and used to constructed cDNA libraries for sequencing with the Illumina NovaSeq 6000 sequencer at Shanghai Majorbio Bio-pharm Biotechnology Co., Ltd. (Shanghai, China). Clean reads were separately aligned to mouse reference genomes by HISAT2. The raw count of genes in each sample were assembled by StringTie. The counts matrix was normalized by DESeq2. Cluster analysis, GO, KEGG, and Gene Set Enrichment Analysis (GSEA) were used to analyze gene expression and related pathways in each group.

### Statistical analysis

Data were analyzed by GraphPad Prism 9.0 software (GraphPad Software, USA). Student’s *t* test was used to compare two groups of data. One-way analysis of variance (ANOVA) was used to compare multiple groups of data. Two-way ANOVA was used to compare data including multiple groups with two or more variation. Data from each experiment were presented as the means ± SD. *P* < 0.05 was considered statistically significant and depicted as follows: *P < 0.05, **P < 0.01.

## Results

### SOAT1 promotes the malignant progression of human HCC

**Fig. 1:**
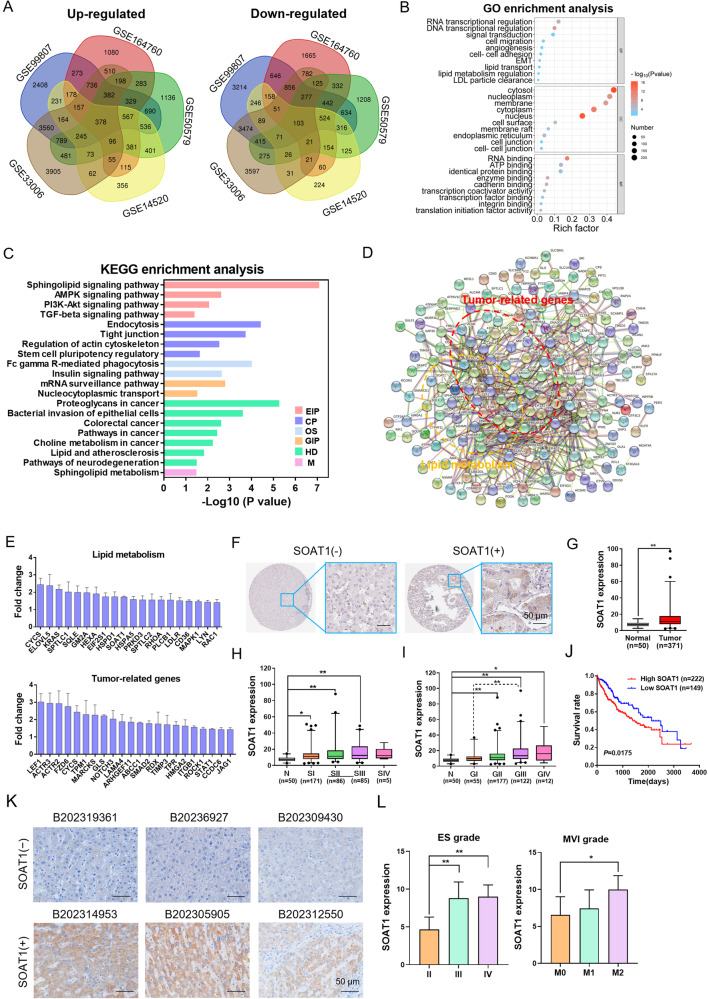
SOAT1 promotes the malignant progression of HCC. **A** Up-regulated and down-regulated differentially expression genes (DEGs) in five mRNA expression profiles. **B** The GO category for DEGs. BP biological process, CC cellular component, MF molecular function. The color represents the *P* value, and the size indicates the enrichment gene number of each pathway. **C** KEGG enrichment pathways of DEGs. EIP Environmental Information Processing, CP Cellular Processes, OS Organismal Systems, GIP Genetic Information Processing, HD Human Diseases, M Metabolism. **D** Protein–protein interaction network of DEGs. **E** Up-regulated gene expression of lipid metabolism and tumor progression. **F** Representative images of IHC staining for SOAT1 of normal liver and HCC tissues cited from The Human Protein Atlas. **G** SOAT1 expression level in normal tissues and HCC tissues based on the TCGA dataset. **H**, **I** Analysis of the SOAT1 expression levels in TCGA HCC samples based on the individual clinical stage (**H**) and pathological grade (**I**). **J** High SOAT1 expression is positively correlated with poor survival (*P* = 0.0175). **K** Representative images of positive and negative SOAT1 expression in different HCC tissues detected by IHC. **L** Analysis of the expression levels of SOAT1 in HCC patient liver tissues based on ES grade and MVI grade.

We selected five GSE profiles about human HCC tissues (including tissues with NASH background) and normal or para-carcinoma tissue to analysis the differential gene and related pathway. A total of 378 up-regulated and 103 down-regulated differentially expressed genes (DEGs) were determined (Fig. 1A). GO enrichment analysis suggested that biological processes (cell migration, angiogenesis, EMT and lipid metabolism regulation), cellular components (cell surface, nucleus, cell junction and endoplasmic reticulum) and molecular functions (RNA binding, transcription factor binding and integrin binding) significantly enriched in HCC tissue (Fig. 1B). KEGG pathway analysis was correlated with lipid metabolism and tumor-related pathway (Fig. 1C). STRING could predict proteins interactions including direct (physical) and indirect (functional) interactions. STRING analysis was performed on DEGs. The results showed that some DEGs are related to lipid metabolism and tumor development (Fig. 1D). In depth, the results showed that tumor related and lipid metabolism-related genes such as cholesterol metabolism genes were increased, including SOAT1 (Fig. 1E). To further explore the clinicopathologically relevant feature of SOAT1, the LIHC data in the Human Protein Atlas and TCGA database was analyzed. The result showed that SOAT1 expression was higher in HCC tissues compared with normal liver tissue (Fig. 1F, G). Besides, SOAT1 expression level positively correlated with individual clinical stage (Fig. 1H) and pathological grade (Fig. 1I). The survival analysis showed that the high SOAT1 mRNA expression level was associated with the poor prognosis in HCC patients (Fig. 1J). We collected 22 cases of HCC tissues and detected SOAT1 expression by immunohistochemistry (IHC). The results showed that SOAT1 exhibited significant high expression in tissue with higher malignancy levels (Fig. 1K). Further analysis showed that the level of SOAT1 expression was positively correlated with the ES grade and MVI grade of HCC (Fig. 1L). Accordingly, these data demonstrated that SOAT1 expression was positively correlated with malignant process.

### SOAT1 promotes the EMT in HCC

**Fig. 2:**
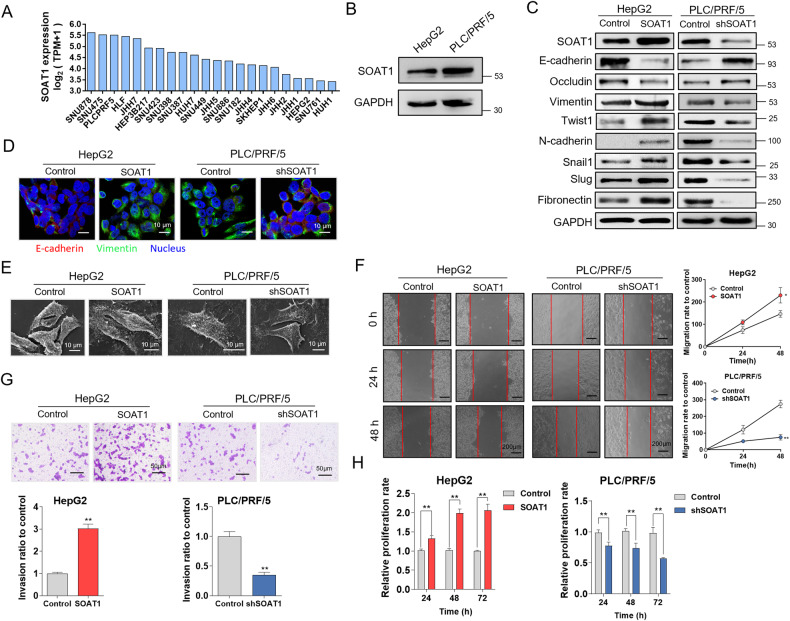
SOAT1 promotes the EMT in HCC cells. **A** SOAT1 expression level in different HCC cell lines cited from CCLE database. **B** Western blot analysis of SOAT1 expression in HepG2 and PLC/PRF/5 cell lines. **C** Western blot analysis of EMT related markers in SOAT1 overexpressed or knocked down cells. **D** Immunofluorescence assay of E-cadherin and Vimentin in cells treated with SOAT1 overexpression or shRNA vectors. **E** Cell phenotype changes under SOAT1 overexpressed or knocked down treatment. **F**, **G** Migration (**F**) and invasion (**G**) of HepG2 cells transfected with SOAT1 or PLC/PRF/5 cells transfected with shSOAT1. **H** Cell proliferation under SOAT1 overexpressed or knocked down treatment.

SOAT1 expression was observed in different HCC cell lines through CCLE public data analysis (Fig. 2A). The SOAT1 expression level was detected in HepG2 and PLC/PRF/5 cell lines by western blot (Fig. 2B). To evaluate the efficacy of SOAT1 expression on EMT, SOAT1 was overexpressed in HepG2 and knocked down in PLC/PRF/5. Western blot analysis revealed that SOAT1 remarkably increased Vimentin, Twist1, N-cadherin, Snail1, Slug, and Fibronectin, but decreased E-cadherin and Occludin expression in HepG2 cells, and the opposite results were observed in PLC/PRF/5 cells with SOAT1 knocked down (Fig. 2C). Immunofluorescence analysis suggested that E-cadherin expression was reduced, and Vimentin expression was increased in SOAT1-overexpressing HepG2 cells. Conversely, SOAT1 knockdown increased E-cadherin expression but decreased Vimentin expression (Fig. 2D). Besides, the influence of SOAT1 in cell phenotypes was observed through scanning electron microscopy. The pseudopodia of cellular surface were increased, and cell morphology changed from epithelial phenotype to mesenchymal phenotype in SOAT1-overexpressing cells and epithelioid phenotype was resumed after the SOAT1 knocked down (Fig. 2E). Moreover, overexpressed SOAT1 promoted cell migration and invasion, which was inhibited by SOAT1 knockdown (Fig. 2F, G). The cell proliferation of HepG2 and PLC/PRF/5 cells with SOAT1 overexpressed or knocked down was detected for 24, 48, and 72 h by CCK-8 assay. The results showed that SOAT1 overexpression promoted cell proliferation, whereas SOAT1 knockdown inhibit cell proliferation (Fig. 2H). These results demonstrated that SOAT1 promoted EMT in HCC cells.

### SOAT1 induces EMT via regulating cholesterol metabolism

**Fig. 3:**
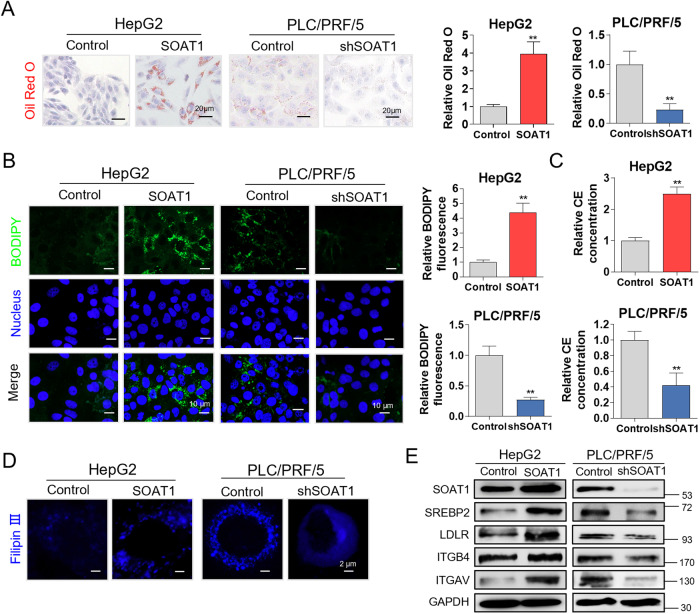
SOAT1 induces the EMT via regulating cholesterol metabolism. **A**, **B** Oil red O (**A**) and BODIPY 493/503 (**B**) staining of lipid droplets in SOAT1 overexpressed HepG2 cells and SOAT1 knocked down PLC/PRF/5 cells. The relative Oil red O and intensity of BODIPY493/503 were analyzed. **C** SOAT1 increased accumulation of cholesterol esters. **D** Cellular cholesterol distribution by Filipin III staining. **E** Western blot analysis of SOAT1, SREBP2, LDLR, ITGAV, and ITGB4 expression levels under SOAT1 overexpression or knockdown.

SOAT1 was an essential modulator of cholesterol esters formation. To further investigate how SOAT1 mediates EMT, lipid droplets were stained by Oil red O and BODIPY493/503. We observed that lipid droplets highly increased in SOAT1 overexpressed groups and decreased in SOAT1 silenced groups (Fig. 3A). Consistent results were acquired through BODIPY staining in HepG2 and PLC/PRF/5 cells with SOAT1 overexpressed or knocked down (Fig. 3B). The accumulation of cholesterol esters was observed in SOAT1-overexpressed cells, and cholesterol esters concentration decreased in SOAT1 knockdown cells (Fig. 3C). SOAT1 overexpression promoted cholesterol accumulation in plasmalemma, and SOAT1 knockdown contributed to the accumulation of intracellular cholesterol (Fig. 3D). Furthermore, we found that SOAT1 overexpression increased the expression level of SREBP2 and LDLR. Besides, the expression level of ITGB4 and ITGAV, integrin related to tumor metastasis, were also increased. The contrary results were obtained in SOAT1 knocked down cells (Fig. 3E). These results revealed that SOAT1 may promote EMT by regulating cholesterol level.

### Nootkatone alleviates cholesterol metabolism disorder by targeting SOAT1

**Fig. 4:**
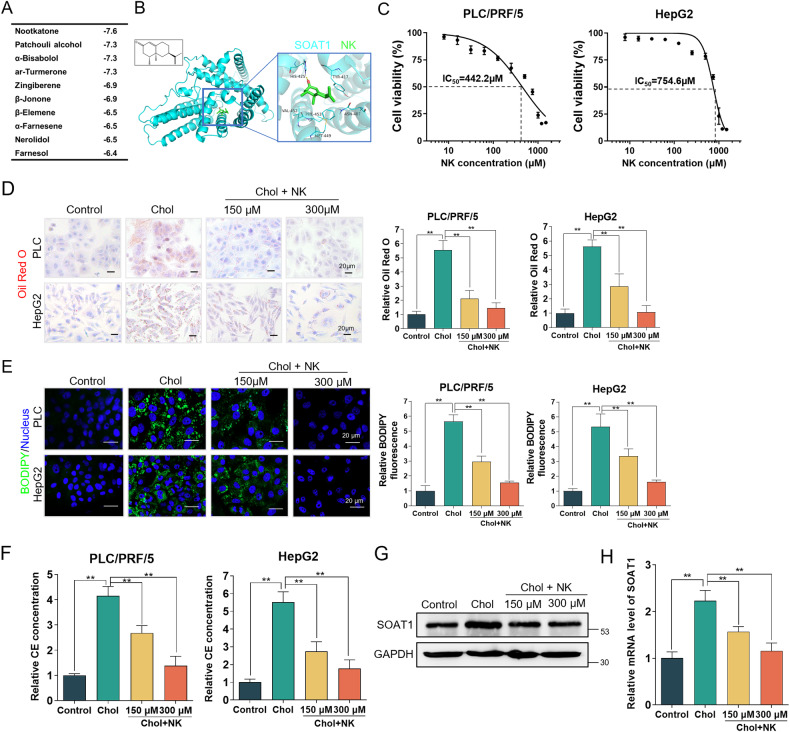
Nootkatone alleviates cholesterol metabolism disorder by targeting SOAT1. **A** Prediction docking score between small-molecule compounds and SOAT1. **B** Predicted interaction of nootkatone with cavity residues of SOAT1. **C** Cell viability of HCC cells with nootkatone treatment for 48 h. **D**, **E** Micrographs of Oil red O (**D**) and BODIPY (**E**) staining of lipid droplets in PLC/PRF/5 induced with cholesterol (200 μg/mL) for 24 h and then treated with nootkatone (150 and 300 µM) for 48 h. **F** Nootkatone decreased the content of cholesterol esters in different groups. **G**, **H** Expression of SOAT1 in different groups.

To investigate SOAT1 inhibitor, some compounds from Medicine Food Homology Compound Library were selected. Molecular docking analysis demonstrated that nootkatone displayed highest affinity with the catalytic pocket of SOAT1 protein (Fig. 4A). The detailed docking result of nootkatone and SOAT1 is shown in Fig. 4B. The results showed that nootkatone had close contact with His425, Tyr417, Met449, Val452, Phe453, and Asn487 of SOAT1, in which the carbonyl oxygen of nootkatone might form a hydrogen bond with His425 of SOAT1 (Fig. 4B). Nootkatone inhibited cell viability of HepG2 and PLC/PRF/5 cell lines (Fig. 4C). And, Nootkatone has no significant effect on the proliferation of normal liver cells (Fig. S1). To determine whether nootkatone is responsible for maintaining cholesterol homeostasis, HepG2 and PLC/PRF/5 cells were cultured in medium containing cholesterol (200 μg/mL) for 24 h and then treated with nootkatone (150 and 300 μM). The Oil red O and BODIPY493/503 staining results indicated that cholesterol promoted lipid accumulation in HCC cells, while nootkatone alleviated accumulation of lipid droplets induced by cholesterol (Fig. 4D, E). Cholesterol esters concentration markedly increased in the cholesterol-induced cells, which was reversed by nootkatone (Fig. 4F). Moreover, nootkatone decreased the effect of protein and mRNA expression level of SOAT1 mediated by cholesterol (Fig. 4G, H). Together, these results confirmed that nootkatone played a crucial role in regulating cholesterol metabolism via targeting SOAT1.

### Nootkatone inhibits the EMT of HCC by targeting SOAT1

**Fig. 5:**
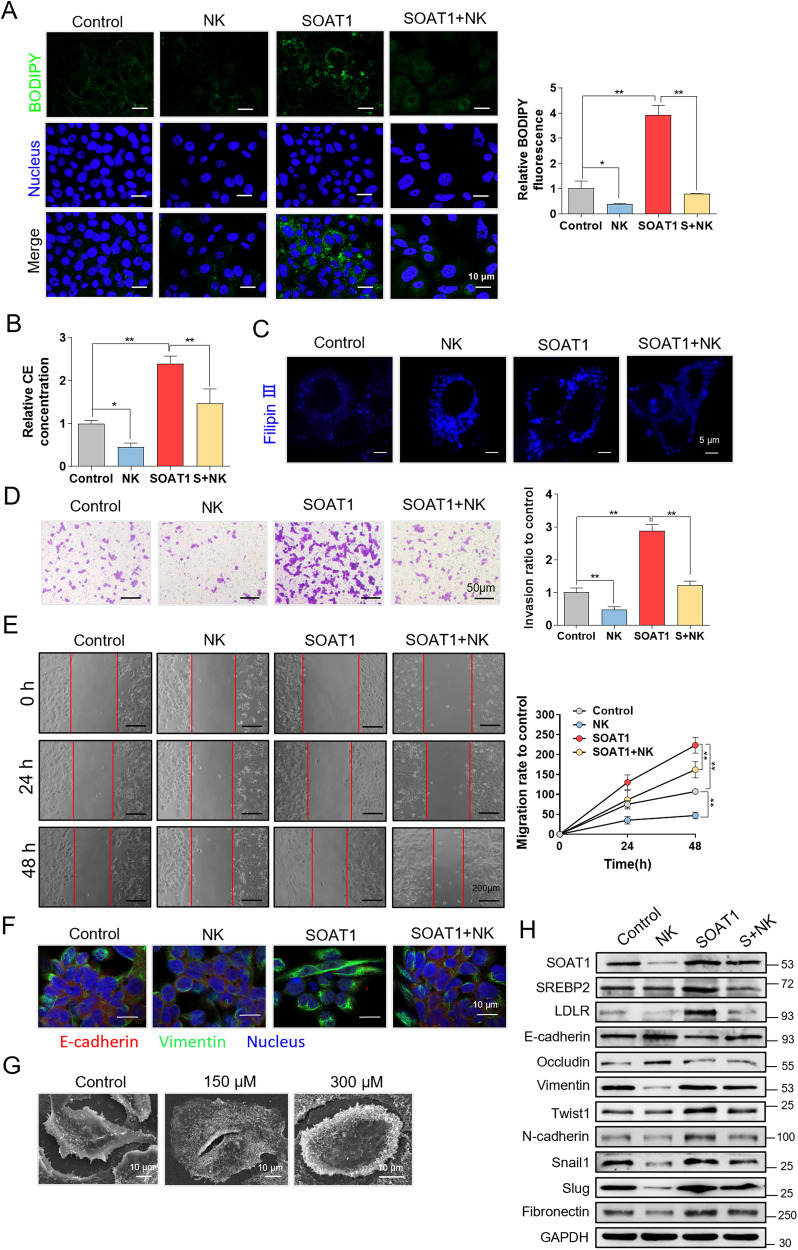
Nootkatone inhibits EMT of HCC by targeting SOAT1. **A** BODIPY493/503 staining of lipid droplets in Control, NK, SOAT1 and SOAT1 + NK groups. **B** Content of cholesterol esters in different groups. **C** Cholesterol distribution was determined by Filipin III staining. **D**, **E** Invasion (**D**) and migration (**E**) of PLC/PRF/5 cells with different treatments. **F** Immunofluorescence assay of E-cadherin and Vimentin of cells in different group. **G** Cell phenotype under nootkatone treatment with different concentration (150 and 300 µM). **H** Western blot analysis of SOAT1, SREBP2, LDLR, E-cadherin, Occludin, Vimentin, Twist1, N-cadherin, Snail1, Slug, and Fibronectin expression level in different groups.

To further investigate whether nootkatone inhibits EMT by targeting SOAT1, rescue experiments were conducted. BODIPY493/503 staining results showed that nootkatone (300 μM) counteracted lipid droplets accumulation facilitated by SOAT1 (Fig. 5A), which was verified by the cholesterol esters content of cells in different groups (Fig. 5B). SOAT1 overexpression contributed to cholesterol accumulation in cell membrane. Nootkatone promoted intracellular cholesterol accumulation, which is similar to the result of SOAT1 knocked down. Also, nootkatone counteracted membrane cholesterol accumulation induced by SOAT1 overexpression (Fig. 5C). Nootkatone inhibited cell invasion and migration, and abolished the migration and invasion mediated by SOAT1 overexpression (Fig. 5D, E). IF analysis suggested that nootkatone decreased Vimentin and increased E-cadherin expression, and eliminates the effect of Vimentin and E-cadherin expression mediated by SOAT1 (Fig. 5F). SEM was used to observe the effect of nootkatone on cell morphology, and the results showed that nootkatone inhibited the mesenchymal phenotype of cells (Fig. 5G). Western blot analysis further proved that nootkatone eliminated the effect of SREBP2, LDLR, E-cadherin, Occludin, Vimentin, Twist1, N-cadherin, Snail1, Slug, and Fibronectin expression mediated by SOAT1 overexpression (Fig. 5H). Overall, these results demonstrated that nootkatone inhibited EMT by targeting SOAT1.

### Nootkatone suppresses the oncogenic and metastatic effects of SOAT1 in vivo

**Fig. 6:**
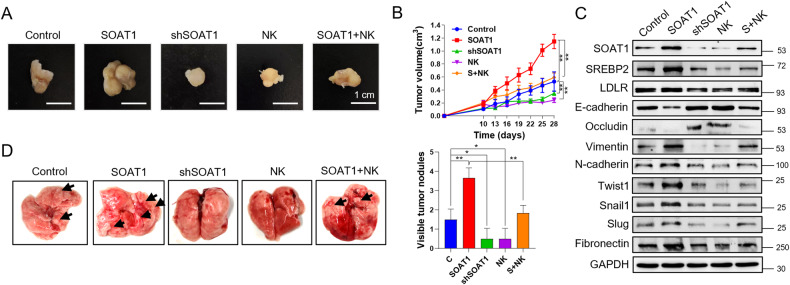
Nootkatone suppresses the oncogenic and metastatic effects of SOAT1 in vivo. **A** Representative images of subcutaneous tumor xenografts in Control, SOAT1, shSOAT1, NK and SOAT1 + NK groups. **B** Tumor volume in different groups. **C** WB analysis of SOAT1, SREBP2, LDLR, E-cadherin, Occludin, Vimentin, Twist1, N-cadherin, Snail1, Slug, and Fibronectin expression levels in tumor tissue of different groups. **D** Visible metastatic nodules on the surface of lungs in different groups.

To validate the oncogenic effects of SOAT1 in vivo, nude mice were subcutaneously implanted PLC/PRF/5 cells to establish xenograft model. Compared with the mice in Control group, SOAT1 promoted the tumor growth, and the opposite results were obtained after silencing SOAT1 expression. Nootkatone administration inhibited tumor growth and eliminated the stimulative roles of SOAT1 on tumor growth (Fig. 6A, B). Moreover, the protein expression of SOAT1, SREBP2, LDLR and EMT related markers in xenograft tumors were detected. The expression level of SREBP2, LDLR and mesenchymal markers (Vimentin, Twist1, N-cadherin, Snail1, Slug, and Fibronectin) were increased in SOAT1 overexpressed group and decreased in SOAT1 silenced group, whereas the E-cadherin and Occludin showed the contrary trend. Nootkatone restored the up-regulation of SREBP2, LDLR, Vimentin, Twist1, N-cadherin, Snail1, Slug, and Fibronectin and down-regulation of E-cadherin and Occludin induced by SOAT1 (Fig. 6C). The results of lung metastasis experiments showed that the number of lung metastatic nodules increased in mice injected with SOAT1 overexpressed PLC cells, whereas decreased in mice of shSOAT1 and NK groups. Nootkatone administration reduced the effect of SOAT1 on lung metastatic nodules (Fig. 6D). These results manifested that SOAT1 played oncogenic and metastatic role in HCC, which was suppressed by nootkatone treatment.

### Nootkatone suppresses tumorigenesis and development of NAFLD-HCC mice

**Fig. 7:**
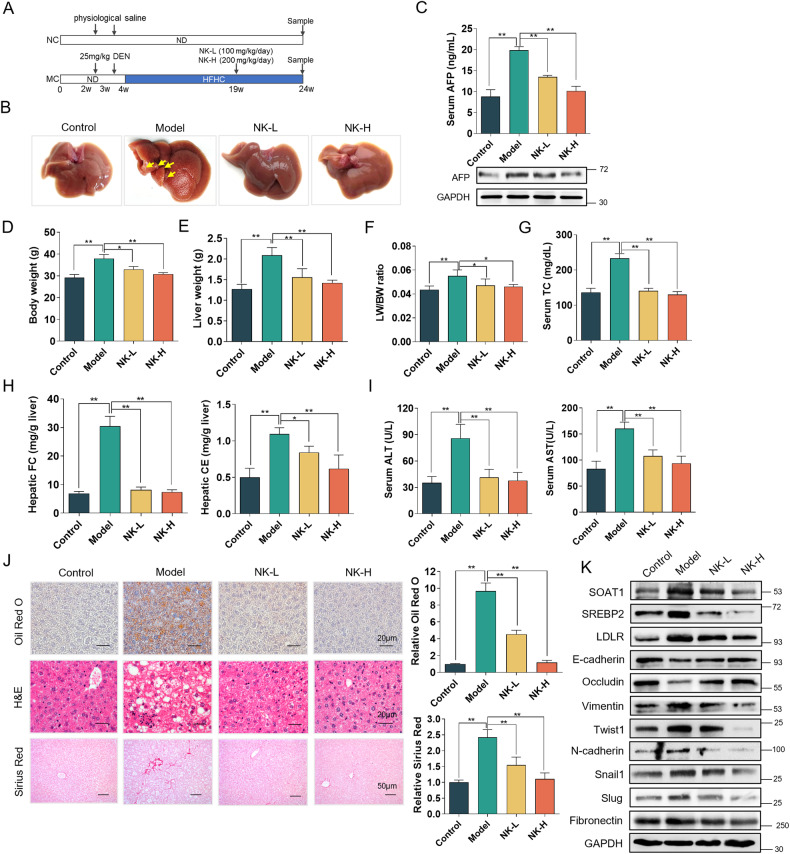
Nootkatone suppresses tumorigenesis and development of NAFLD-HCC mice. **A** Schematic illustration of experimental procedure. **B** Representative macroscopic images of liver in Control, Model, NK-L, and NK-H groups. **C** AFP expression in serum and liver tissue of mice in different groups. **D** Body weight of mice in different groups. **E**, **F** Liver weight (**E**) and liver weight-to-body weight ratio (**F**). **G** Serum TC level in four groups. **H** Contents of hepatic free cholesterol and cholesterol esters in four different groups. **I** Serum ALT and AST level in different groups. **J** Morphological observations of the liver and liver tissue with Oil red O, H&E and Sirius red staining. The relative Oil red O and Sirius red were obtained through the Image J Pro software. **K** The protein expression level of SOAT1, SREBP2, LDLR, E-cadherin, Occludin, Vimentin, Twist1, N-cadherin, Snail1, Slug, and Fibronectin expression in liver tissue of mice in different groups.

Classical mice model of NAFLD-HCC induced by DEN injection and HFHC feeding was established to verify that nootkatone inhibits EMT by targeting SOAT1 in NAFLD-HCC mice (Fig. 7A). The respective liver macroscopic suggested that compared to the control group, liver of model group mice displayed uneven surface and tumor nodules (yellow arrow), indicating that NAFLD-HCC model was successfully conducted. Nootkatone obviously improved HFHC + DEN-induced NAFLD-HCC in mice (Fig. 7B). Alpha fetoprotein (AFP) is one of the most widely accepted detection markers of HCC. The results of AFP detection in serum and liver tissue showed that the liver of the model group mice underwent carcinogenesis, and nootkatone administration reduced AFP levels (Fig. 7C). Besides, mice in model group displayed enhanced body weight, liver weight, and liver-to-body weight ratio (LW/BW ratio), which were improved by nootkatone (Fig. 7D–F). Serum total cholesterol was significantly increased in model mice, which were recovered by nootkatone treatment (Fig. 7G). In parallel, hepatic free cholesterol and cholesterol ester content were increased in model mice, whereas nootkatone significantly reduced the accumulation of hepatic free cholesterol and cholesterol esters (Fig. 7H). Serum ALT and AST levels were significantly increased in model mice compared with that in control group, which were decreased by nootkatone supplementation (Fig. 7I). Lipid accumulation in liver was evaluated through Oil red O staining. The wide distribution of large lipid droplets was observed in liver of model mice, whereas nootkatone administration decreased lipid droplets distribution in liver. H&E staining results showed that DEN + HFHC-feeding resulted in balloon-like structures and lipid deposition in liver tissue. Notably, nootkatone supplementation effectively decreased the hepatocellular ballooning and lipid deposition in liver. Moreover, model mice exhibited fibrotic injury with increased collagen distribution, while nootkatone reduced hepatic collagen deposition to ameliorate liver fibrosis (Fig. 7J). Furthermore, the expression of SOAT1, SREBP2, LDLR and Vimentin, Twist1, N-cadherin, Snail1, Slug, and Fibronectin was increased, while E-cadherin and Occludin expression was decreased in liver tissues of model mice. Nootkatone markedly reversed the expression in a dose-dependent manner (Fig. 7K). IHC staining results suggested that compared with the control group, reduced E-cadherin and increased vimentin expression were observed in liver of model mice, while nootkatone administration reversed their expression (Fig. S2). These results indicate that the trend of NAFLD to HCC transition induced by DEN + HFHC significantly increases SOAT1 expression in liver tissue, resulting in the disruption of cholesterol homeostasis, and nootkatone can alleviate liver lesions.

**Fig. 8:**
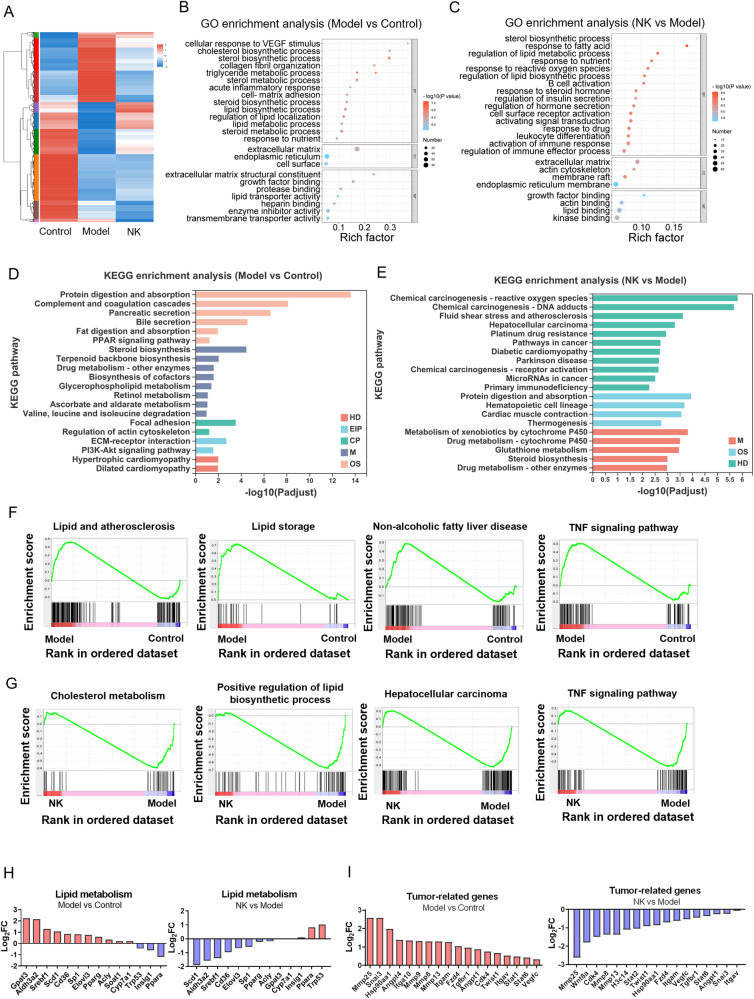
mRNA-seq analysis verifies that nootkatone inhibits tumorigenesis and development of NAFLD-HCC mice. **A** Heatmap of the hierarchical clustering DEGs from liver tissue in different groups (log_2_FC > 2, *P* < 0.01). Red represents up-regulated genes and blue represents down-regulated genes. **B**, **C** GO enrichment of DEGs in Model vs Control (**B**) and NK vs Model (**C**) groups. BP biological process, CC cellular component, MF molecular function. The color represents the *P* value, and the size indicates the enrichment gene number of each pathway. **D**, **E** KEGG pathway enrichment of DEGs in Model vs Control (**D**) and NK vs Model (**E**) groups. HD Human Diseases, EIP Environmental Information Processing, CP Cellular Processes, M Metabolism, OS Organismal Systems. **F**, **G** GSEA of Model vs Control (**F**) and NK vs Model (**G**) groups. **H** Up-regulated and down-regulated DEGs of lipid metabolism in Model vs Control and NK vs Model groups. **I** Up-regulated DEGs associated with tumorigenesis and development in Model vs Control and NK vs Model groups.

To further explore the effects of nootkatone on cholesterol metabolism and oncogenesis in the progression of NAFLD to HCC, mRNA-Seq was performed with liver tissue of NAFLD-HCC mice. The differently expression genes (DEGs) in Control, Model and NK group are represented in Fig. 8A. GO analyzed the DGEs between every two groups (Model vs Control and NK vs Model). In Model vs Control group, lipid metabolism related function (sterol synthetic, cholesterol biosynthetic process, and lipid localization) and tumor-related process (collagen fibril organization, cell matrix adhesion, growth factor binding, and extracellular matrix) obviously enriched (Fig. 8B). Nootkatone specially regulated lipid metabolism (response to fatty acid, lipid biosynthetic process and lipid metabolic process), tumor-related process (extracellular matrix, actin cytoskeleton, and growth factor binding) and immune response (B cell activation, leukocyte differentiation and immune effector process) (Fig. 8C). KEGG pathway enrichment analysis indicated that DEGs in Model vs Control group enriched in protein digestion and absorption, lipid metabolism related pathway (fat digestion and absorption, steroid biosynthesis) and tumorigenesis and development related pathway (PPAR signaling pathway, focal adhesion and PI3K/Akt signaling pathway) (Fig. 8D). It is noteworthy that the DGEs in NK vs Model group enriched in pathways including HCC, pathways in cancer, drug resistance and metabolism (Fig. 8E). Moreover, GSEA results suggested that DEN + HFHC induction accelerated lipid and atherosclerosis, lipid storage, NAFLD, and TNF signaling pathway (Fig. 8F). Nootkatone suppressed cholesterol metabolism, lipid biosynthetic process, HCC, and TNF signaling pathway (Fig. 8G). In detail, the upregulated and downregulated lipid metabolism related genes were analyzed, and nootkatone effectively maintained lipid metabolism homeostasis (Fig. 8H). In addition, DEN + HFHC-fed resulted in the increased expression of representative genes related to tumorigenesis and development, whereas nootkatone administration inhibited tumor-related genes expression (Fig. 8I). In order to verify the mRNA sequencing results, we verified the expression of cholesterol metabolism-related genes and tumor-related genes in the liver tissue of the Control, Model, NK-L, and NK-H groups by qRT-PCR. Compared with the Control group, the mRNA expression of *Srebf2*, *Scap*, *Hmgcr*, *Soat1*, *Npc1*, *Snai3*, *Twist1*, *Itgav*, *Vegfc*, and *Tgfbr1* has higher expression in model group, indicating that there is lipid metabolism disorder in model mice. Nootkatone administration could inhibited the mRNA expression to maintain lipid metabolism homeostasis (Fig. S3). Taken together, these results demonstrated that DEN + HFHC drove the process of NAFLD-HCC, while nootkatone could inhibit the process.

## Discussion

EMT is a critical process contributing to cancer progression and metastasis [39]. Increasing studies suggest that EMT is inextricably linked to cholesterol metabolism [4, 40]. SOAT1, a key cholesterol esterification enzyme, can transform supernumerary intracellular cholesterol into cholesterol esters storing in lipid droplets. Previous study has found that SOAT1 is a diagnostic marker and therapeutic target for HCC [30]. Tumor suppressor P53 represses ubiquitin-specific peptidase 19 (USP19) which deubiquitinate and stabilize SOAT1, thereby inhibiting hepatocarcinogenesis [41]. However, the underlying mechanism that SOAT1 promotes EMT is unclear in HCC. In this study, TCGA database and clinical specimen information analysis showed that high SOAT1 expression is a risk factor for survival of HCC patients. In vitro, we found that SOAT1 decreased the expression of epithelium protein marker (E-cadherin and Occludin) and increased the expression of mesenchymal protein (Vimentin, Twist1, N-cadherin, Snail1, Slug, and Fibronectin), promoting invasion and migration of HCC cells. Of note, SOAT2 shares high homologous to SOAT1 [42] but has lower expression in HCC than normal liver tissues, which needs further research.

Abnormal cholesterol metabolism accelerated the development of HCC. SREBP2, is a master transcriptional regulator of cholesterol biosynthesis pathway [43]. SCAP could regulated SREBP2 from the endoplasmic reticulum (ER) by sensing and responding to ER cholesterol fluctuations [44]. When SOAT1 were overexpressed, ER membrane cholesterol is depleted, the SCAP-SREBP2 complex is sorted into COPII vesicles and moves from the ER to the Golgi for proteolytic activation of SREBP2, promoting the expression of LDLR which related to cholesterol uptake [10]. In previous studies, cholesterol synthesis mediated by SREBP2 promotes the caveolin-dependent endocytosis of integrin β1, contributing to pathological angiogenesis [45]. Besides, the cholesterol level of the plasma membrane regulates receptors on the plasma membrane, including integrins [46, 47]. Targeting cholesterol/lipid raft/integrin β3/FAK pathway overcomes EMT-associated drug resistance [48]. In our study, the results indicated that SOAT1 overexpression promoted the accumulation of lipid droplets and cholesterol esters. Moreover, SOAT1 overexpression promoted cholesterol synthesis and uptake, accelerating the accumulation of cholesterol in plasma membrane, which enhanced the integrins (ITGAV and ITGB4) expression. When SOAT1 was knocked down, the process of cholesterol into cholesterol ester was blocked, leading to intracellular cholesterol accumulation. The xenograft tumor and lung metastasis model in vivo revealed that SOAT1 accelerated tumor growth and metastasis.

NAFLD is closely linked to HCC development [49]. Cholesterol or dietary cholesterol facilitates hepatocarcinogenesis [50, 51]. Increased serum lipid level and hepatic steatosis are remarkable characteristic of NAFLD [52]. AFP, as a serum biomarker and tumor antigen, is the most widely accepted detection indicator in HCC [53]. Hepatic free cholesterol and cholesterol ester accumulation is another feature in HCC [14, 41]. We conducted NAFLD-HCC mice model to determine the mechanism that SOAT1 induced hepatocarcinogenesis. DEN + HFHC feeding resulted in tumor nodules and high level of AFP in serum and liver. Moreover, SOAT1 and mesenchymal markers were highly expressed in model mice, while epithelium markers were decreased. Besides, abnormal cholesterol metabolism was observed in NAFLD-HCC mice, which was presented as increased cholesterol level in serum and liver tissue, increased lipid droplets accumulation, higher protein and mRNA expression of cholesterol metabolism-related genes. These data demonstrated that SOAT1 was involved in the process of tumor occurrence.

Notably, despite cholesterol metabolism is a frequent pathway for the antitumor drugs, there are no specific targets and drugs for clinical use. Previous study indicated that SOAT1 exhibits high expression in the S-III subtype of HCC, and knocked down SOAT1 inhibit tumor development [46]. Currently, some SOAT1 inhibitors have been found, including avasmibe [7], pactimibe [54], PD-122301 (nevanimibe) [42], and CI-976 [55]. Nootkatone, a natural sesquiterpene ketone, was extracted from grapefruit peel. Previews studies have confirmed that nootkatone ameliorates liver fibrosis and inhibits tumor cells proliferation, such colorectal cancer and non-small-cell lung cancer [27–29]. However, the therapeutic effect of nootkatone on HCC has not been reported. The molecular docking results showed that the binding site between nootkatone and SOAT1 (His425, Tyr417, Met449, Val452, Phe453, Asn487 residues) is close to the binding site between Oleoyl-CoA, a cholesterol esterified substrate, and the cytosolic tunnel TM7 of SOAT1. Therefore, we speculate that nootkatone, as a possible competitive inhibitor, might inhibit SOAT1 activity by hindering the loading of substrate into the catalytic center. The binding mechanism between nootkatone and SOAT1 needs further exploration. We further investigated that nootkatone inhibited EMT by targeting SOAT1-induced cholesterol metabolism in vitro. In addition, nootkatone treatment significantly inhibited the xenograft tumor growth and pulmonary metastasis via targeting SOAT1. Moreover, nootkatone supplementation reduced the expression of SOAT1, alleviated hepatic steatosis and pathological injuries damage, which suppressed tumorigenesis and development of NAFLD-HCC. So, we hold that nootkatone might inhibit EMT by targeting SOAT1.

Our studies confirmed that SOAT1 accelerated the EMT procession of HCC by regulating cholesterol metabolism in vitro and in vivo. Nootkatone, a natural medicine food homology compound, inhibited EMT via targeting SOAT1. These findings reveal that SOAT1 is a potential anti-tumor metastasis target in HCC, providing the reference for targeted cholesterol metabolism to cure HCC. Importantly, our investigation provides new insights into the anti-cancer mechanism of sesquiterpene and demonstrates that the natural compound nootkatone might be valuable as a potential drug for cancer therapy.

### Supplementary information

Supplementary material

Original Western Blot

## Data Availability

The data that support the findings of this study are available from this manuscript and the corresponding author upon reasonable request.
