# Supplementary material for: SOAT1 regulates cholesterol metabolism to induce EMT in hepatocellular carcinoma
Source: Cell Death Dis. 2024 May 9;15(5):325. doi: 10.1038/s41419-024-06711-9 (PMC11082151; doi:10.1038/s41419-024-06711-9)
Supplement: Supplementary file 1 — Supplementary material [file 41419_2024_6711_MOESM1_ESM.docx]

**Supplementary data**


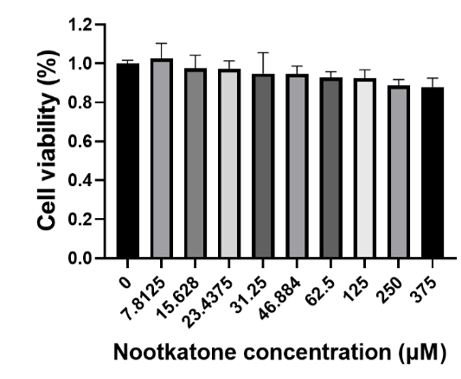


Fig. S1 Cell viability of THLE-2 with different concentration nootkatone treatment for 48h.

**
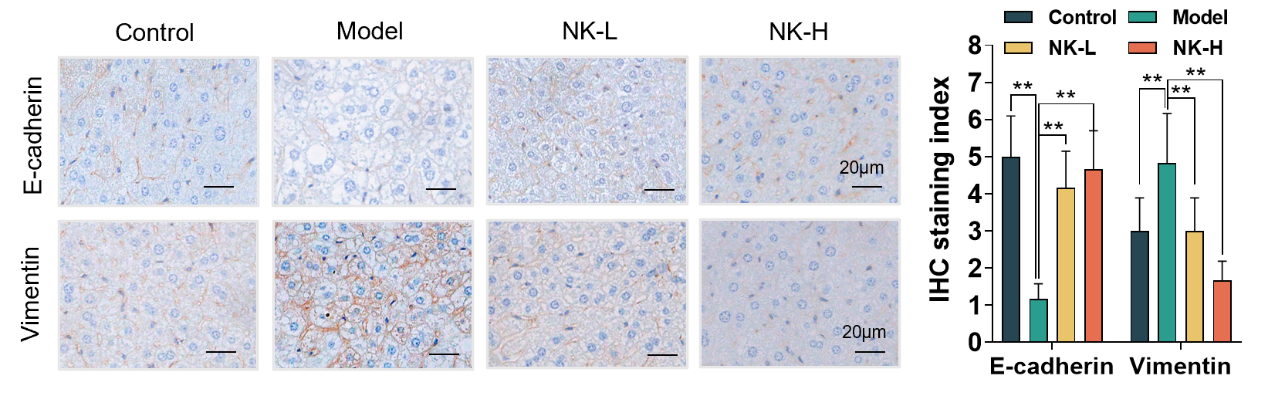
**

Fig. S2 E-cadherin and Vimentin expression in the liver tissues of different groups were analyzed via IHC.


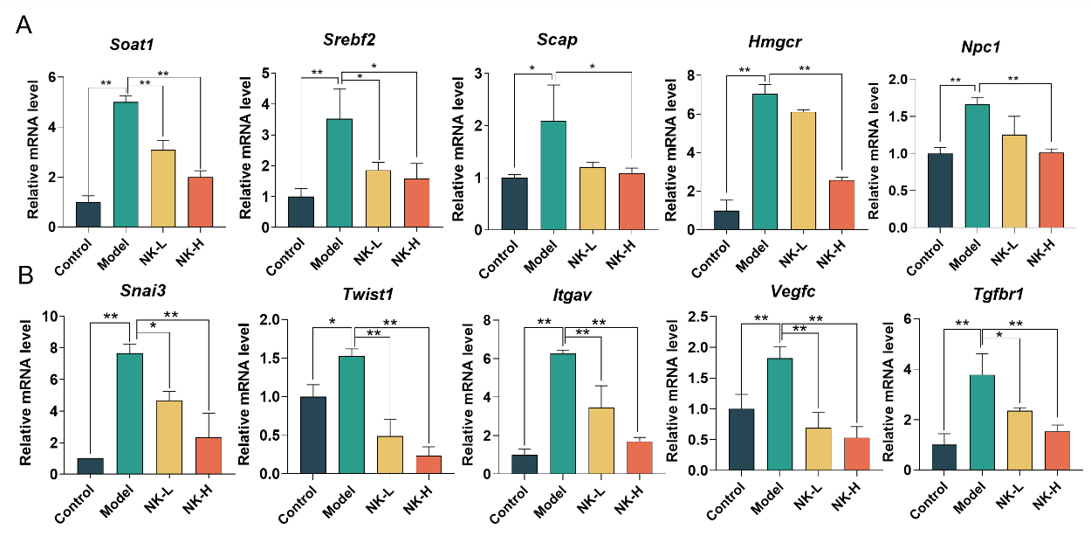


Fig. S3 Relative mRNA gene expressions of (A) lipid metabolism (*Soat1*, *Srebf2*, *Scap*, *Hmgcr*, and *Npc1*) and (B) tumor-related genes (*Snai3*, *Twist1*, *Itgav*, *Vegfc*, and *Tgfbr1*) in liver tissue of mice from different groups. * *P*<0.05, ***P*<0.01.
