# Supplementary figures and images for: SOAT1 regulates cholesterol metabolism to induce EMT in hepatocellular carcinoma
Source: Cell Death Dis. 2024 May 9;15(5):325. doi: 10.1038/s41419-024-06711-9 (PMC11082151; doi:10.1038/s41419-024-06711-9)

Fig.2B

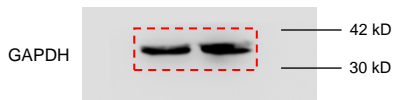

Fig.2C

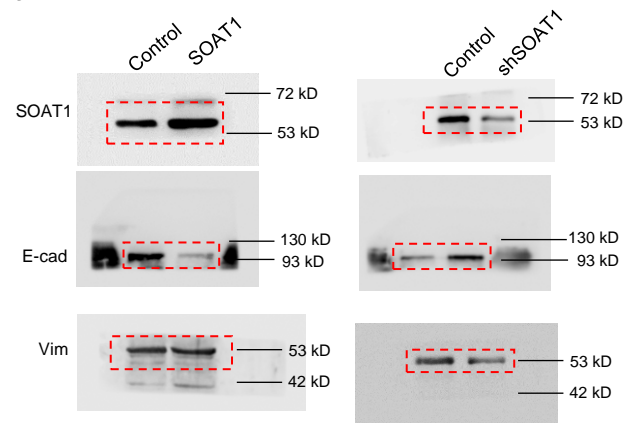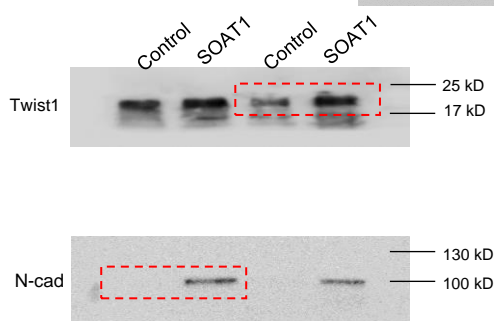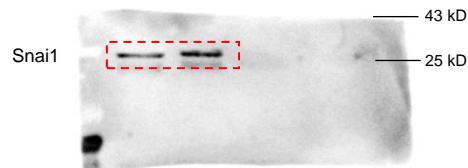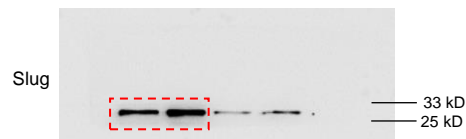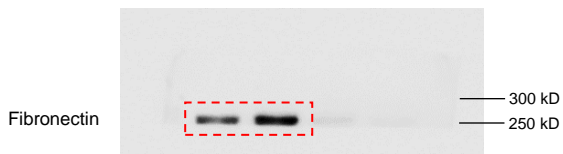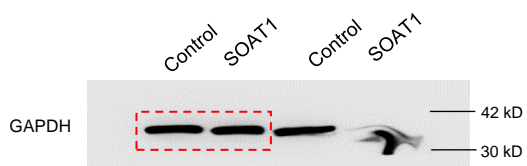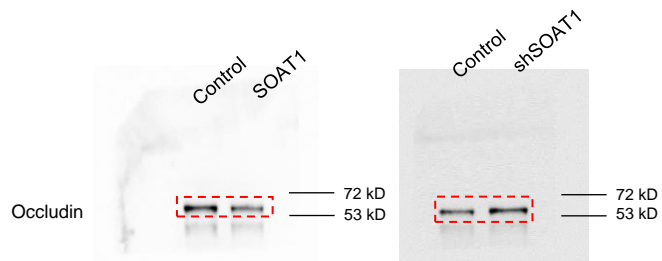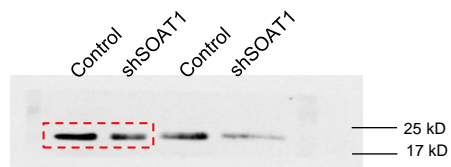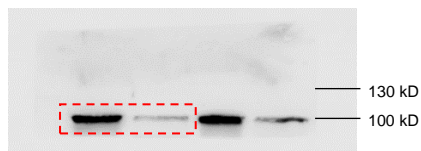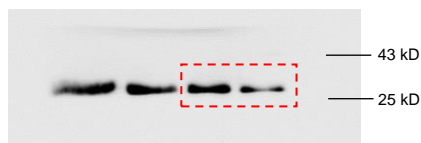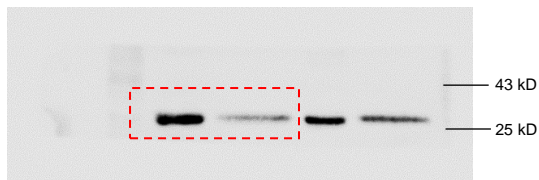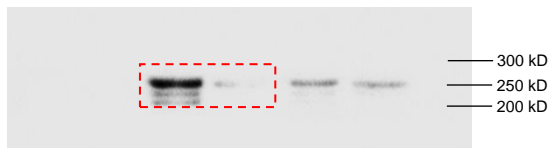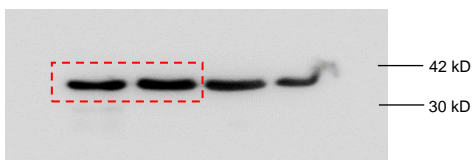

Fig.3E

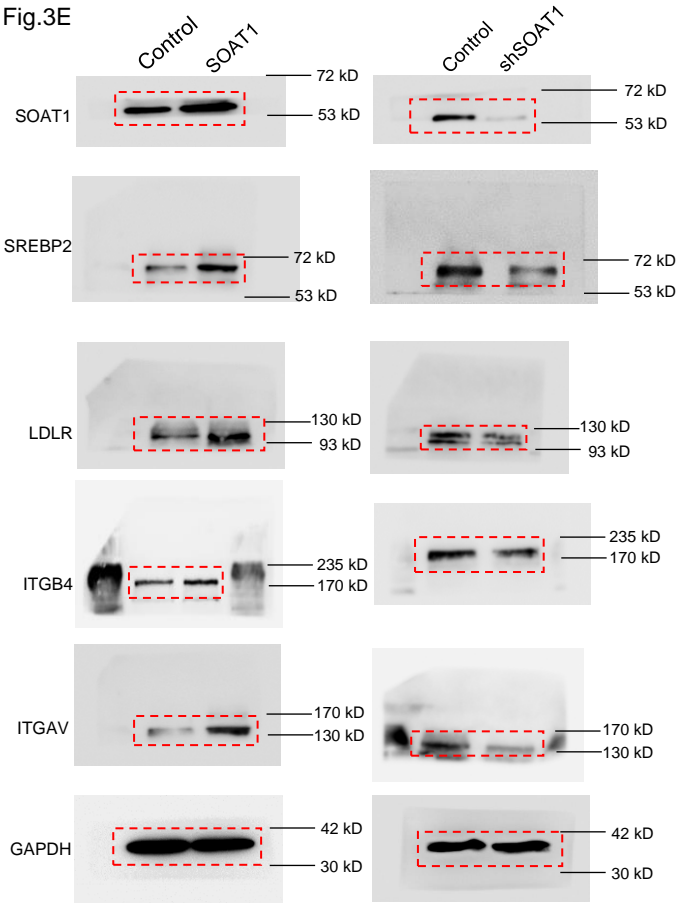

Fig.4 G

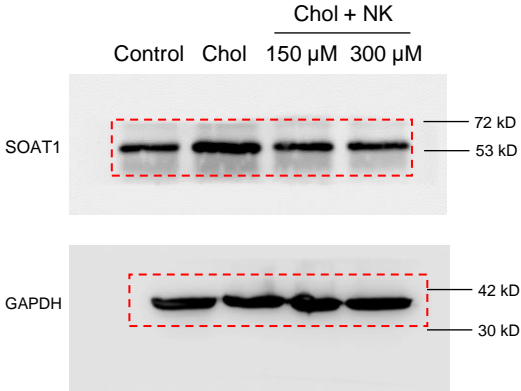

Fig.5G

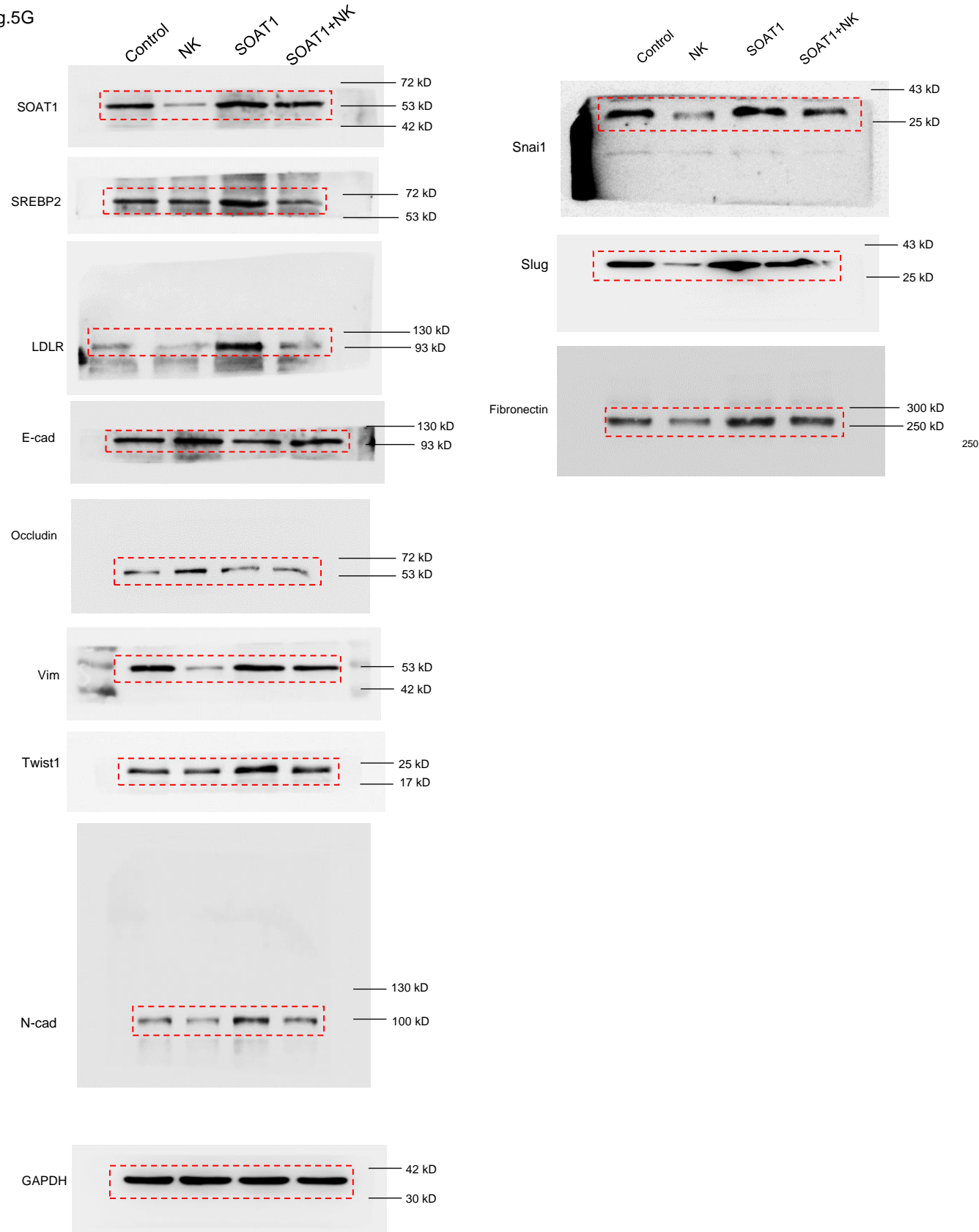

Fig.6C

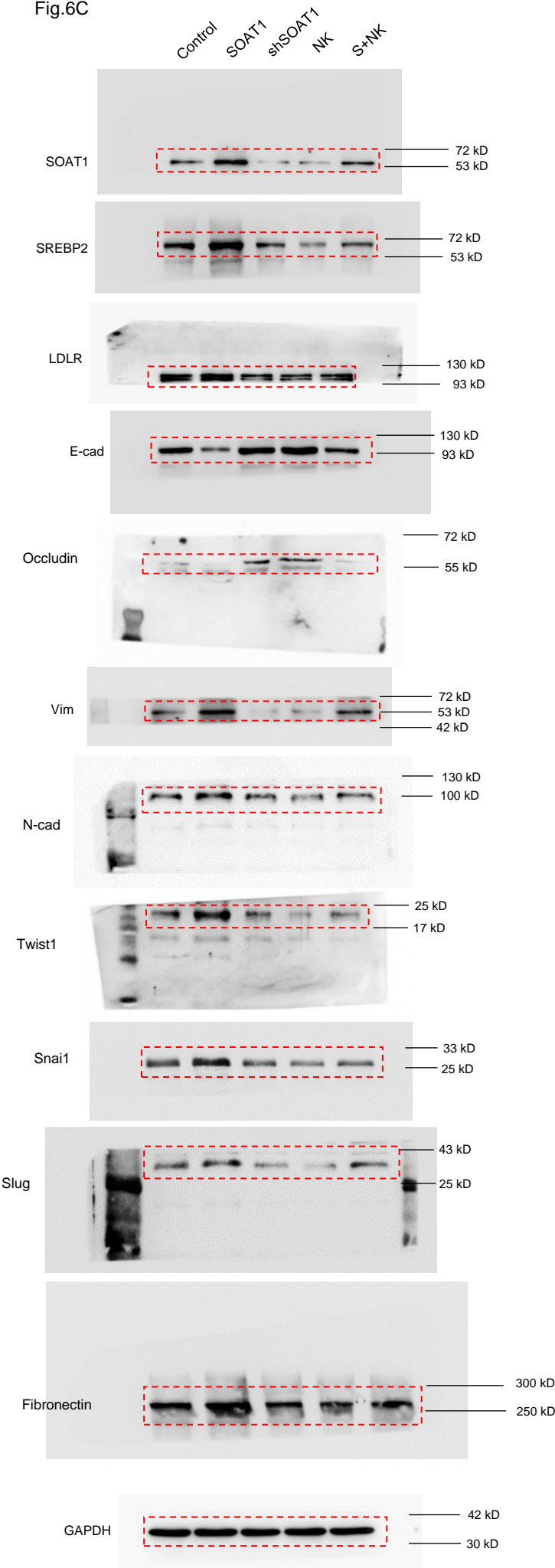

Fig. 7I

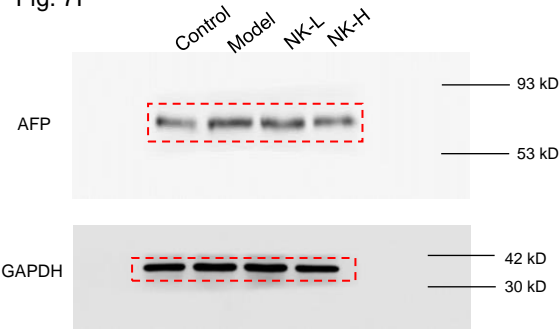

Fig.7 J

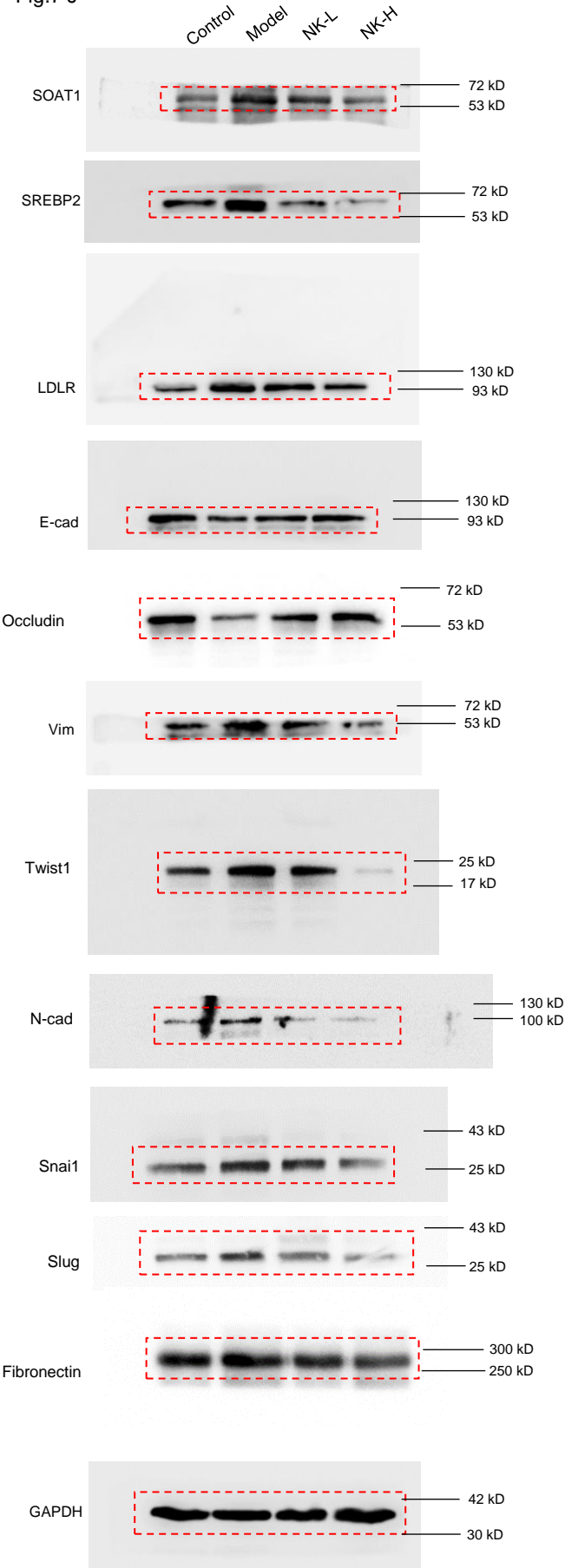

Supplement: Supplementary file 2 — Original Western Blot [file 41419_2024_6711_MOESM2_ESM.pdf]
